# Construction of an oligometastatic prediction model for nasopharyngeal carcinoma patients based on pathomics features and dynamic multi-swarm particle swarm optimization support vector machine

**DOI:** 10.3389/fonc.2025.1589919

**Published:** 2025-06-19

**Authors:** Yunfei Li, Dongni Zhang, Yiren Wang, Yiheng Hu, Zhongjian Wen, Cheng Yang, Ping Zhou, Wen-Hui Cheng

**Affiliations:** ^1^ Department of Oncology, The Affiliated Hospital of Southwest Medical University, Luzhou, China; ^2^ School of Nursing, Southwest Medical University, Luzhou, China; ^3^ Wound Healing Basic Research and Clinical Applications Key Laboratory of Luzhou, School of Nursing, Southwest Medical University, Luzhou, China; ^4^ Department of Medical Imaging, Southwest Medical University, Luzhou, China; ^5^ School of Clinical Medicine, Southwest Medical University, Luzhou, China; ^6^ Department of Radiology, The Affiliated Hospital of Southwest Medical University, Luzhou, China; ^7^ Department of Ophthalmology, The Affiliated Hospital of Southwest Medical University, Luzhou, China

**Keywords:** machine learning, prediction model, metastases, nasopharyngeal carcinoma, pathomics, support vector machine

## Abstract

**Objective:**

This study aimed to develop a risk prediction model for post-treatment oligometastasis in nasopharyngeal carcinoma (NPC) by integrating pathomics features and an improved Support vector machine (SVM) algorithm, offering precise early decision support.

**Methods:**

This study retrospectively included 462 NPC patients, without or with oligometastasis defined by ESTRO/EORTC criteria. Whole-slide images were scanned, and three representative H&E-stained regions were selected for pathomics feature extraction via CellProfiler software. Features screened by intraclass correlation coefficient, Mann-Whitney U test, Spearman correlation, minimum redundancy maximum relevance, and Least absolute shrinkage and selection operator regression. Based on these screened features, three models were built: Dynamic Multi-Swarm Particle Swarm Optimization SVM (DMS-PSO-SVM), Particle Swarm Optimization SVM (PSO-SVM), and a standard SVM. Model training and hyperparameter tuning were conducted on the training set (n=369), followed by evaluation on a validation set (n=93).

**Results:**

6 pathomics features were screened as important features. DMS-PSO-SVM yielded superior performance, with training-set AUC=0.880 and validation-set AUC=0.866, consistently outperforming both PSO-SVM (AUC=0.721) and standard SVM (AUC=0.718). Calibration curves showed good agreement for DMS-PSO-SVM (P>0.05) but indicated miscalibration in the standard SVM (P<0.05). Decision curve analysis further demonstrated that DMS-PSO-SVM offered higher net benefit across a wide range of risk thresholds.

**Conclusion:**

Incorporating pathomics and DMS-PSO optimization significantly improved NPC oligometastasis prediction. This model showed high discriminative ability, calibration, and clinical utility, suggesting that pathomics and machine learning-based strategies could aid early recognition of high-risk patients and inform individualized treatment approaches. A demo of the DMS-PSO-SVM modeling algorithm code used in this study can be found on Github (https://github.com/Edward-E-S-Wang/DMS-PSO-SVM).

## 1 Introduction

Nasopharyngeal carcinoma (NPC) is a malignant tumor originating from the nasopharyngeal mucosal epithelium and exhibits distinct geographic and ethnic differences. It is primarily found in southern China and Southeast Asian countries, with 47.7% of new cases worldwide occurring in China (1, 2). Clinically, NPC often presents with cervical lymph node enlargement, tinnitus, and epistaxis, and it progresses rapidly, commonly leading to distant metastasis that significantly impacts patients’ quality of life and prognosis (3). Although radiotherapy combined with chemotherapy has become the standard treatment for NPC and has effectively increased overall survival rates, distant metastasis remains a major factor affecting long-term survival and cure rates (4). The five-year overall survival rate for patients with oligometastasis is much lower than for those without distant metastasis; moreover, when multiple organ metastases occur, both overall survival and quality of life markedly decline (5). In clinical practice, accurately identifying and assessing the risk of oligometastasis at an early stage is challenging. Once metastasis occurs, treatment strategies and prognosis become considerably more complex, and existing conventional staging systems show limitations in accurately predicting the possibility of oligometastasis before treatment. Some studies have indicated that patients initially diagnosed with oligometastatic NPC may achieve long-term survival and a favorable prognosis through adequate systemic treatment and high-dose radiotherapy. Therefore, early detection and prediction of oligometastasis in NPC has become a crucial issue in clinical settings (6).

In recent years, radiomics analysis based on medical imaging modalities such as MRI, CT, and PET/CT has commonly been used in research for NPC predictive models (7). However, radiomics has limited capacity to capture tumor biological phenotypes, tumor microenvironment characteristics, and intratumoral heterogeneity, thereby restricting the accuracy of predicting treatment response and the risk of subsequent recurrence or metastasis (8). The pathomics concept, which leverages quantitative features extracted from digital pathology slides, has garnered increasing attention. By performing high-throughput feature extraction and quantitative analysis on scanned histopathological slides, pathomics can capture subtle changes in the tumor and its microenvironment from multiple dimensions such as morphology, texture, and spatial distribution (9). Compared with conventional imaging, pathology constitutes the most direct histological evidence in diagnosing cancer and evaluating its malignancy. Pathomics-related studies in various solid tumors have demonstrated initial success in risk stratification, recurrence prediction, and efficacy assessment (10, 11). This further supports the rationale and potential for incorporating pathomics into oligometastatic prediction for NPC. Moreover, no existing studies have constructed a predictive model for distant metastasis in NPC using pathomics features.

Machine learning algorithms have been extensively applied to radiomics, genomics, and pathomics in studies involving different “-omics” domains (12–14). Their ability to perform large-scale data mining and multidimensional data integration has shown promising predictive performance in NPC-related clinical events (15). In early identification of NPC oligometastasis and the development of individualized treatment strategies, combining pathomics features with machine learning techniques can not only enable efficient selection of pathomics information from tissue slides but also allow for comprehensive integration and screening of multidimensional data, thus further improving the model’s predictive accuracy and reliability. Therefore, this study aims to extract pathomics features from pre-treatment histopathological slides of NPC patients and use machine learning methods to build a pathomics-based model for predicting post-treatment oligometastasis in NPC. The goal is to provide clinical decision support for physicians in order to improve patients’ quality of life.

## 2 Methods

### 2.1 Patients

This study included 462 nasopharyngeal carcinoma (NPC) patients treated at the Affiliated Hospital of Southwest Medical University between January 2017 and January 2024. Inclusion criteria were: (1) NPC confirmed by pathological biopsy; (2) Clear pathological slide images; (3) Complete baseline data; (4) NPC patients who received treatment according to NCCN guidelines at this hospital before oligometastasis occurred; (5) Patients who developed oligometastases more than six months after tumor treatment; (6) Patients who did not develop oligometastasis or multiple metastases for more than six months after treatment; (7) Patients aged 18 years or above. Exclusion criteria were: (1) Missing or damaged pathological images; (2) Patients lacking complete baseline clinical data; (3) Patients with other primary malignant tumors or multiple metastases; (4) Patients who developed oligometastasis during or before receiving treatment.

In this study, according to the consensus published by ESTRO and EORTC, the diagnostic criteria for oligometastasis are: (1) ≤5 metastatic sites and ≤2 metastatic organs; (2) Metastatic lesions confirmed by pathological or imaging examinations (6, 16).

**Figure 1:**
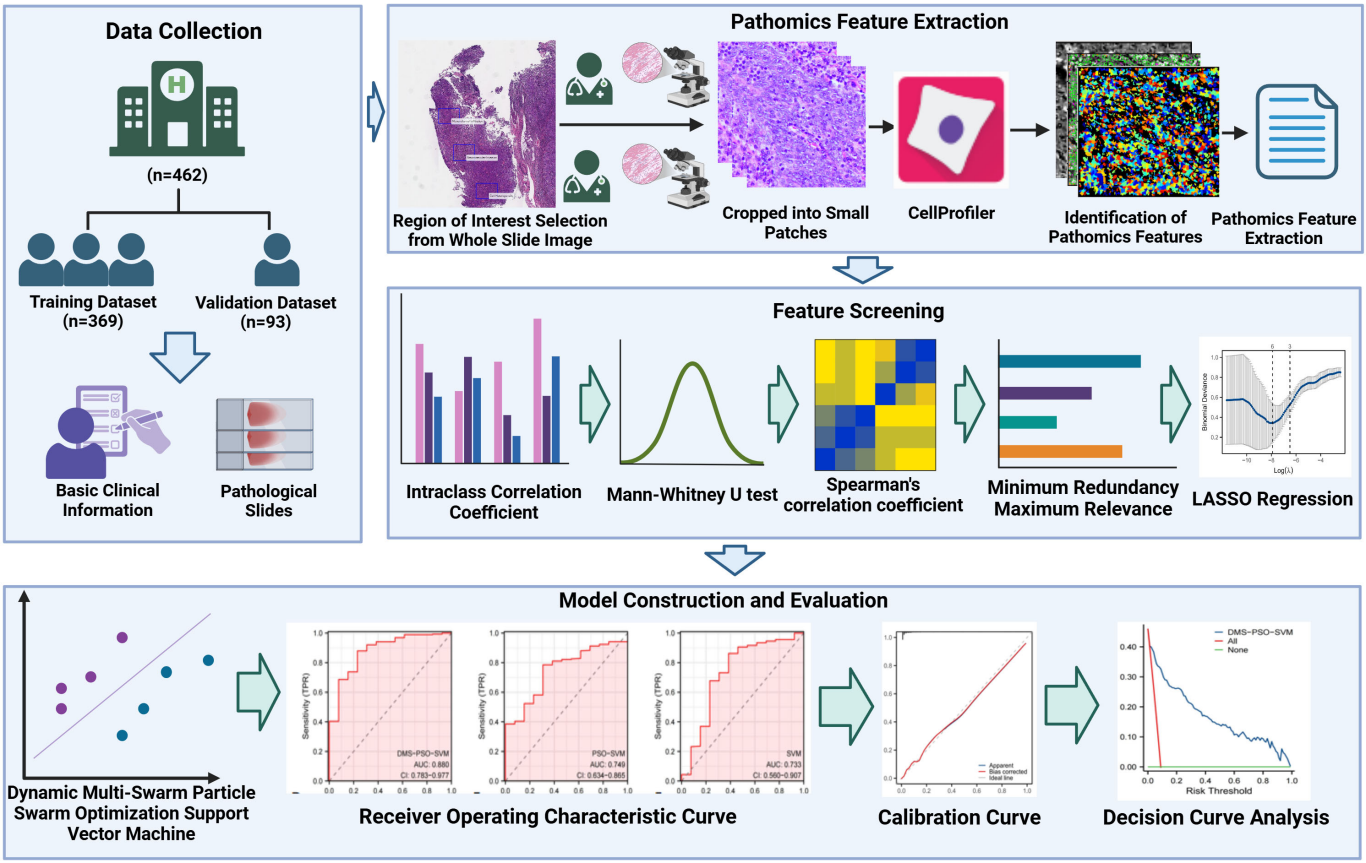
Flowchart of this study.

All patients (n=462) were randomly split into a training set (n=369) and a validation set (n=93) at an 8:2 ratio. The training set was used for subsequent pathomics feature selection and model construction, and the validation set was used to evaluate model performance. This study was approved by the Ethics Committee of the Affiliated Hospital of Southwest Medical University (KY2021023). Due to the retrospective nature of the study, informed consent from patients was waived. The study was conducted in accordance with the Declaration of Helsinki (2013 Revision). A flowchart of this study is shown in **Figure 1**.

### 2.2 Pathology slides acquisition and pathomics feature extraction

All biopsy specimens obtained via needle aspiration were immersed in 10% formalin solution for 4 hours. They were then embedded in paraffin blocks and sectioned at 4 μm intervals, followed by hematoxylin-eosin (H&E) staining for pathological evaluation. Whole slide images (WSIs) of these pathological sections were acquired through a digital slide scanner (KFBio KF-PRO-020, Jiangfeng Bio Co. Ltd., China). Two pathologists, each with ≥5 years of experience in diagnosing pathological slides, enlarged the WSIs to 40x to select regions of diagnostic value. The selection criteria were as follows: (1) stromal infiltration—tumor invasion into adjacent connective tissue; (2) neurovascular invasion—tumor cells invading nearby nerve fibers or blood vessels; and (3) cellular heterogeneity—areas showing high cellular diversity in shape, size, and nuclear atypia with unclear cell boundaries (17). Each pathologist independently collected three typical non-overlapping regions of 1280×960 pixels. In case of disagreement, a senior pathologist with ≥10 years of diagnostic experience made the final determination. Finally, all images were non-overlapping and cropped to 512×512 patches for subsequent in-depth analysis.

Quantitative pathomics features were extracted from the selected patches using CellProfiler software (version 4.0.7). The “Unmix Colors” module was used to separate H&E-stained images into grayscale hematoxylin and eosin channels. In addition, the “ColorToGray” module was employed to convert H&E-stained images into grayscale. Feature extraction comprised three steps. First, primary pathomics features were extracted from the original grayscale image, including blur features, intensity features, and threshold features. Second, separated H&E images were processed via “IdentifyPrimaryObjects” and “IdentifySecondaryObjects” to identify and extract higher-order quantitative image features such as shape, size, texture, and pixel intensity distribution (18, 19). Third, for each whole slide image (WSI), the mean feature values from its three patches were calculated for subsequent analysis.

### 2.3 Feature screening

All extracted pathomics features were normalized to a range of 0 to 1 via min-max normalization, addressing batch-to-batch staining differences in H&E slides. Feature screening involved five steps. First, the intraclass correlation coefficient (ICC) was used to assess feature reproducibility, and features with high reproducibility (ICC > 0.75) were retained (20). Second, the Mann-Whitney U test was performed to identify pathomics features significantly associated with the dependent variable (P < 0.05) for further analysis (21). Third, Spearman correlation was used to evaluate inter-feature correlations; if the correlation coefficient between two features exceeded 0.9, only one was kept (22). After these steps, the minimum redundancy maximum relevance (mRMR) method was employed to maximize the correlation between features and the classification variable while minimizing inter-feature correlations, thereby refining the feature set (23). Lastly, the Least absolute shrinkage and selection operator (LASSO) regression algorithm was used to identify key pathomics features. LASSO regression determines an optimal penalty parameter λ to select the best feature combination, assigning a regression coefficient to each important feature (24). This study used lambda.min and five-fold cross-validation, where the chosen λ produced the lowest cross-validation error. The pathomics features selected via LASSO were considered significant and were used for subsequent predictive model construction.

### 2.4 Model construction

After the final retained pathomics features were determined through the screening process, these features were incorporated into a support vector machine (SVM) model for binary classification to predict oligometastatic risk. In the SVM model, both the penalty factor *C* and the width parameter *γ* of the RBF kernel significantly influence the shape of the decision boundary and the model’s generalization capability. However, relying solely on manual or grid search in a high-dimensional parameter space can cause the search to become trapped in local optima or require excessive computational costs. To address this issue, Dynamic multi-swarm particle swarm optimization (DMS-PSO) was introduced to optimize SVM parameters globally, while k-fold cross-validation was employed within the training set to assess the classification performance of each candidate parameter set (25).

In the training set, the pathomics features retained by LASSO were first constructed into a matrix *X*, and the corresponding label vector *Y* recorded whether each patient had developed oligometastases. The tunable hyperparameters of SVM were mapped to the two-dimensional position of particles in the swarm:


$$x_{i}=(C_{i},\gamma_{i})$$

Where $C_{i}$ corresponds to the penalty factor, and $\gamma_{i}$ corresponds to the RBF kernel width. Particle initial positions and velocities were randomly generated within the given interval:


$$\left[C_{min},\;C_{max}]\times [\gamma_{min},\;\gamma_{max}\right]$$

Subsequently, all particles were divided into several smaller sub-swarms. Each sub-swarm adopted a local version of the PSO update formula. At iteration step t+1 , the initial velocity of a particle could be obtained by:


$$v_{i,d}(t+1)=\omega \;v_{i,d}(t)+c_{1}r_{1}[pbest_{i,d}-x_{i,d}(t)]+c_{2}r_{2}[lbest_{j,d}-x_{i,d}(t)]$$

Where ω is the inertia weight, ${\text{c}}_{1},{\text{c}}_{2}$ are learning factors, ${\text{r}}_{1},{\text{r}}_{2}$ are random numbers, and ${\text{pbest}}_{\text{i},\text{d}}$ and ${\text{lbest}}_{\text{j},\text{d}}$ denote the particle’s historical best and the sub-swarm’s best solution, respectively, in dimension *d*. Position updates were performed using:


$$x_{i,d}(t+1)=x_{i,d}(t)+v_{i,d}(t+1)$$

If any particle exceeded the specified boundaries after updating, it was clamped to ensure it remained within the legitimate parameter range. After updating within each sub-swarm, all particles were randomly shuffled and reassigned to sub-swarms every fixed iteration cycle *R*, allowing different sub-swarms to rapidly share high-quality solutions at the swarm level and thus avoid local optima.

To evaluate how each particle’s $\left(C_{i},\gamma_{i}\right)$ contributed to model prediction, pathomics features and their corresponding labels in the training set were fed into DMS-PSO-SVM. A 5-fold cross-validation procedure was used, taking AUC as the assessment metric. In order to maximize AUC, the fitness function was defined as  1−AUC , and the algorithm performed a minimization search. If a particle’s fitness was better than its historical best or the sub-swarm’s best,  pbest or lbest was updated accordingly. After multiple iterations, DMS-PSO yielded the current global optimum $\left({\hat{C}}_{\;},{\hat{\gamma }}_{\;}\right)$ , corresponding to the particle with the lowest fitness among all. Once this global best parameter set was determined, a final model was retrained on the training set and evaluated on the validation set to measure its generalization performance. The receiver operating characteristic (ROC) curve’s area under the curve (AUC), accuracy, sensitivity, and specificity were used to assess predictive performance, thereby determining whether the model demonstrated favorable discrimination and robustness in predicting oligometastatic risk in NPC. A calibration curve was employed to compare predicted event probabilities with actual event frequencies for assessing calibration, while decision curve analysis was used to evaluate the clinical net benefit of all models. Additionally, this study compares the prediction performance of the proposed model with four other mainstream CNNs architecture (**Supplementary File 5**).

### 2.5 Model interpretation based on SHAP

To interpret how individual pathomics measurements drive the DMS-PSO-SVM predictions, we applied the SHAP algorithm using the entire training cohort as the reference distribution. The 6 features from all training patients were supplied to the SHAP KernelExplainer together with the final model’s decision function. SHAP values were then computed for each feature in the validation cases and summarized by calculating the mean absolute SHAP value for every feature to establish a global importance ranking. A summary dot plot was also generated to depict each feature’s overall impact on the model output, illustrating both effect size and direction. To demonstrate the model’s behavior at the patient level, we randomly selected four subjects—two who developed oligometastasis and two who remained metastasis-free—and produced force plots showing how each feature shifted their individual risk scores up or down.

### 2.6 Statistical analysis

All statistical analyses in this study were performed using Python (Version 3.10) and R (Version 4.4.1). In the baseline data analysis, categorical variables were compared using the chi-square test or Yates’ continuity-corrected chi-square test. As the numerical variables did not meet normal distribution criteria, the Wilcoxon test was used for two-group comparisons. All tests were two-sided, and a two-tailed P < 0.05 was considered statistically significant. Additionally, the Hosmer-Lemeshow goodness-of-fit test was used to evaluate calibration curves of the models.

## 3 Results

### 3.1 Baseline information

A total of 462 NPC patients were included in this study, and baseline data were collected (**Supplementary File 1**). Among them, 95 patients (20.6%) were diagnosed with oligometastasis, while 367 patients (79.4%) did not experience oligometastasis. The median age for the oligometastatic group was 49 years (IQR: 45–56), whereas the median age for the non-oligometastatic group was 51 years (IQR: 46–57). Moreover, significant differences in N Stage and AJCC Stage were observed between the two groups (P < 0.001). All patients (n=462) were randomly split into a training set (n=369) and a validation set (n=93) in an 8:2 ratio. The training set was used for subsequent pathomics feature screening and model development, while the validation set was employed to assess the performance of the model.

### 3.2 Pathomics feature screening

**Figure 2:**
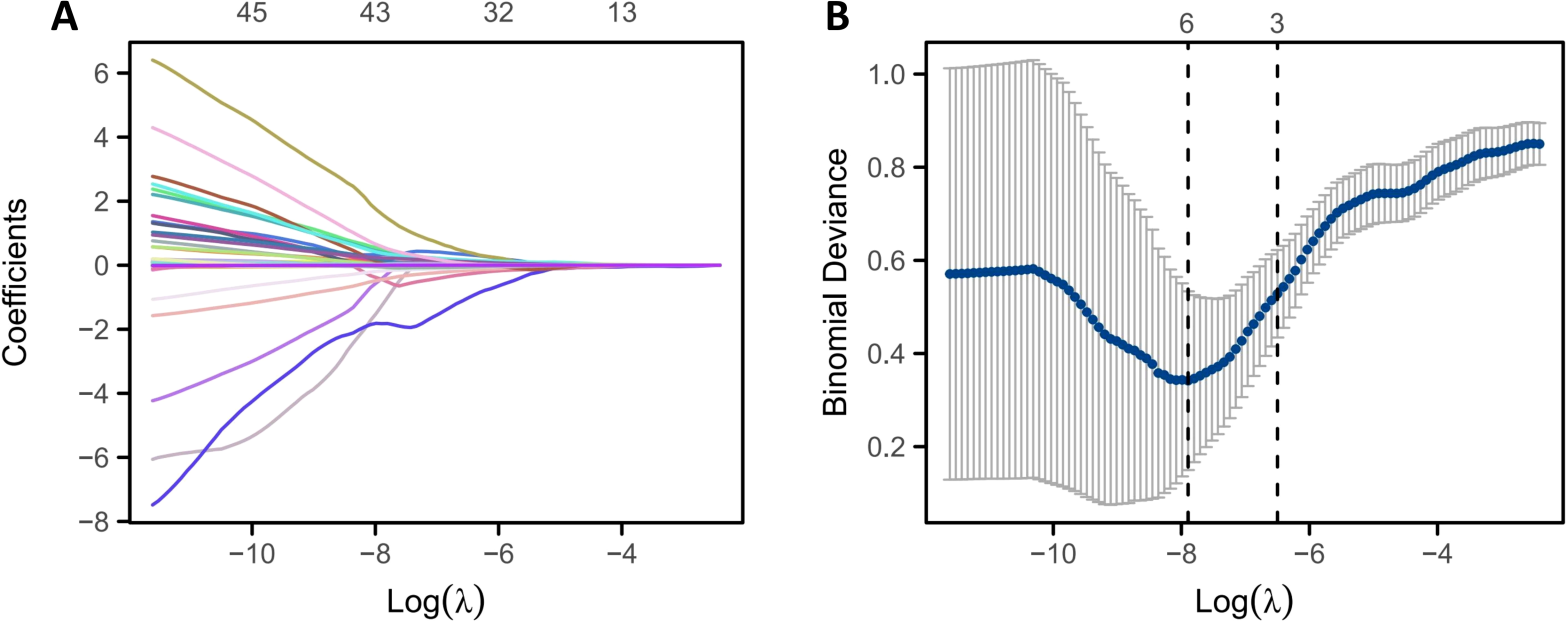
Feature screening based on LASSO regression. **(A)** LASSO regression feature screening trajectory plot; **(B)** LASSO regression feature coefficient screening plot.

A total of 351 pathomics features were obtained from the pathological images in this study. The intraclass correlation coefficients (ICC) for these features ranged from 0.771 to 0.890, indicating good reproducibility of feature extraction. After conducting the U test, 141 pathomics features that were significantly associated with the dependent variable (P < 0.05) were selected. Following Spearman correlation analysis, 108 pathomics features were retained. Subsequently, 76 pathomics features were further filtered using the mRMR algorithm and then entered into LASSO regression for final selection. Employing lambda.min and five-fold cross-validation to determine the optimal penalty parameter, 6 key pathomics features were ultimately identified for model construction (**Figure 2**) (**Supplementary File 2**).

### 3.3 Evaluation of prediction model

**Figure 3:**
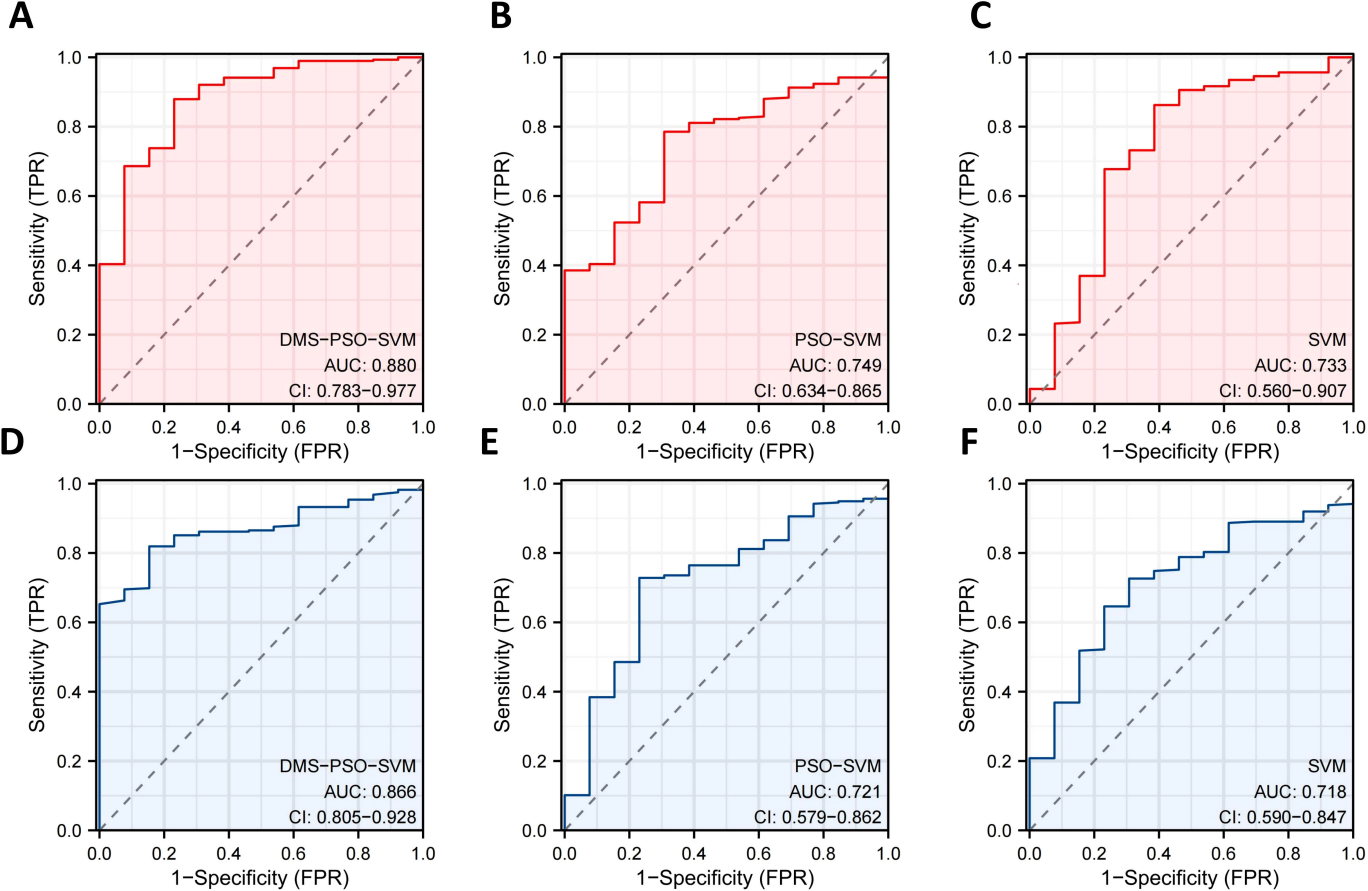
Receiver operating characteristic curve of nasopharyngeal carcinoma oligometastatic prediction model. **(A)** Dynamic multi-swarm particle swarm optimization support vector machine in training set. **(B)** Particle swarm optimization support vector machine in training set. **(C)** Support vector machine in training set. **(D)** Dynamic multi-swarm particle swarm optimization support vector machine in validation set. **(E)** Particle swarm optimization support vector machine in validation set. **(F)** Support vector machine in validation set.

Following feature selection, the six final retained pathomics features were separately used to construct three predictive models—DMS-PSO-SVM, PSO-SVM, and a standard SVM. Based on the principle of maximizing the Youden index in the training set, we determined that the optimal cutoff for the model’s predicted probability is 0.47 (26). Therefore, patients with a predicted risk probability ≥ 0.47 are defined as high-risk (label = 1), and those with a predicted risk probability < 0.47 are defined as low-risk (label = 0). In **Figure 3**, the ROC curves illustrate the classification outcomes and corresponding AUC values on both the training set (**Figures 3A–C**) and validation set (**Figures 3D–F**). After five-fold cross-validation and 138 iterations, the DMS-PSO-SVM model, which employs the dynamic multi-swarm particle swarm optimization for parameter tuning, achieved the best discriminative performance. Its hyperparameter search space and optimal parameter set can be found in **Supplementary File 3**. DMS-PSO-SVM attained an AUC of 0.880 (95% CI: 0.783–0.977) in the training set and 0.866 (95% CI: 0.805–0.928) in the validation set, demonstrating high sensitivity and specificity. In contrast, the AUC values for PSO-SVM in the training and validation sets were 0.749 (95% CI: 0.634–0.865) and 0.721 (95% CI: 0.579–0.862), respectively, while those for the standard SVM were 0.733 (95% CI: 0.560–0.907) and 0.718 (95% CI: 0.590–0.847). The standard SVM in this study used grid search for hyperparameter optimization, and the optimal parameters and search range are provided in **Supplementary File 4**. Furthermore, in the training set, DMS-PSO-SVM achieved an AUC of 0.880, an accuracy of 0.875, a sensitivity of 0.879, and a specificity of 0.769—overall better than PSO-SVM and the standard SVM. Although SVM’s accuracy (0.851) and sensitivity (0.862) were slightly higher than those of PSO-SVM, its specificity was only 0.615, with a relatively low AUC (0.733), indicating a certain degree of overfitting or bias. In the validation set, DMS-PSO-SVM maintained the best discrimination (AUC = 0.866) alongside optimal accuracy (0.820), sensitivity (0.819), and specificity (0.846), whereas PSO-SVM and SVM saw declines in all metrics. Notably, the standard SVM exhibited the lowest AUC, accuracy, sensitivity, and specificity among the three models. These findings further demonstrate that applying a dynamic multi-swarm particle swarm optimization strategy to SVM hyperparameter tuning can enhance the model’s ability to differentiate oligometastatic risk in NPC and yield more stable predictive performance in both the training and validation sets (**Table 1**).

**Table 1:** Evaluation metrics of nasopharyngeal carcinoma oligometastatic prediction model.

Dataset	AUC	Accuracy	Sensitivity	Specificity
Training Dataset				
DMS-PSO-SVM	0.880^bc^	0.875	0.879	0.769
PSO-SVM	0.749^a^	0.781	0.785	0.692
SVM	0.733	0.851	0.862	0.615
Validation Dataset				
DMS-PSO-SVM	0.866^bc^	0.820	0.819	0.846
PSO-SVM	0.721^a^	0.733	0.728	0.769
SVM	0.718	0.724	0.726	0.693

AUC, Area under the curve; DMS-PSO-SVM, Dynamic multi-swarm particle swarm optimization support vector machine; PSO-SVM, Particle swarm optimization support vector machine; SVM, Support vector machine.

Superscripts denote pairwise comparisons of AUC by DeLong’s test;

^a^p> 0.05 versus standard SVM;

^b^p < 0.05 versus standard SVM;

^c^p < 0.05 versus PSO-SVM.

**Figure 4:**
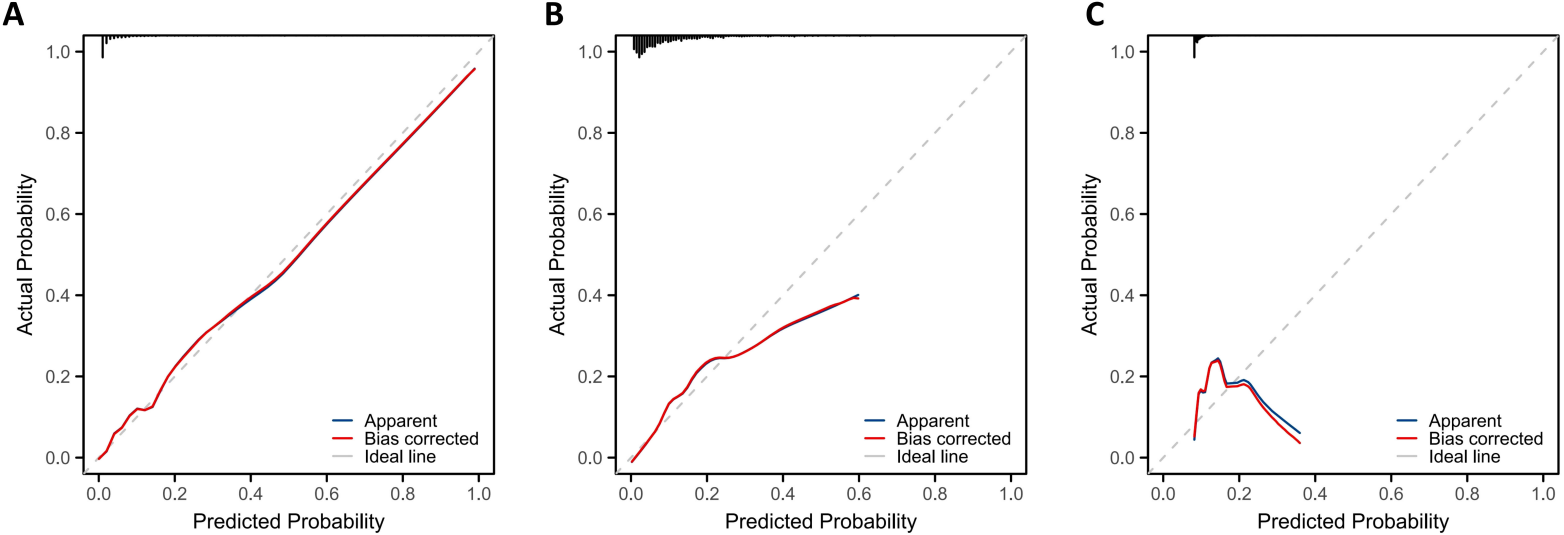
Calibration curve of nasopharyngeal carcinoma oligometastatic prediction Model. **(A)** Dynamic multi-swarm particle swarm optimization support vector machine. **(B)** Particle swarm optimization support vector machine. **(C)** Support vector machine.

This study proceeded to evaluate the calibration of the three models in the validation set via calibration curves (**Figure 4**) and the Hosmer-Lemeshow goodness-of-fit test. Results indicated that the DMS-PSO-SVM (**Figure 4A**) and PSO-SVM (**Figure 4B**) calibration curves closely aligned with the ideal line, with the red bias-corrected curve and the original prediction blue curve both approximating a diagonal, accompanied by a Hosmer-Lemeshow P > 0.05. This suggests good consistency between the models’ predicted probability distributions and actual outcomes. In contrast, the standard SVM (**Figure 4C**) demonstrated a certain level of calibration bias, with the bias-corrected curve deviating considerably from the ideal line and a Hosmer-Lemeshow test result of P < 0.05, implying a significant discrepancy between predicted and observed outcomes.

**Figure 5:**
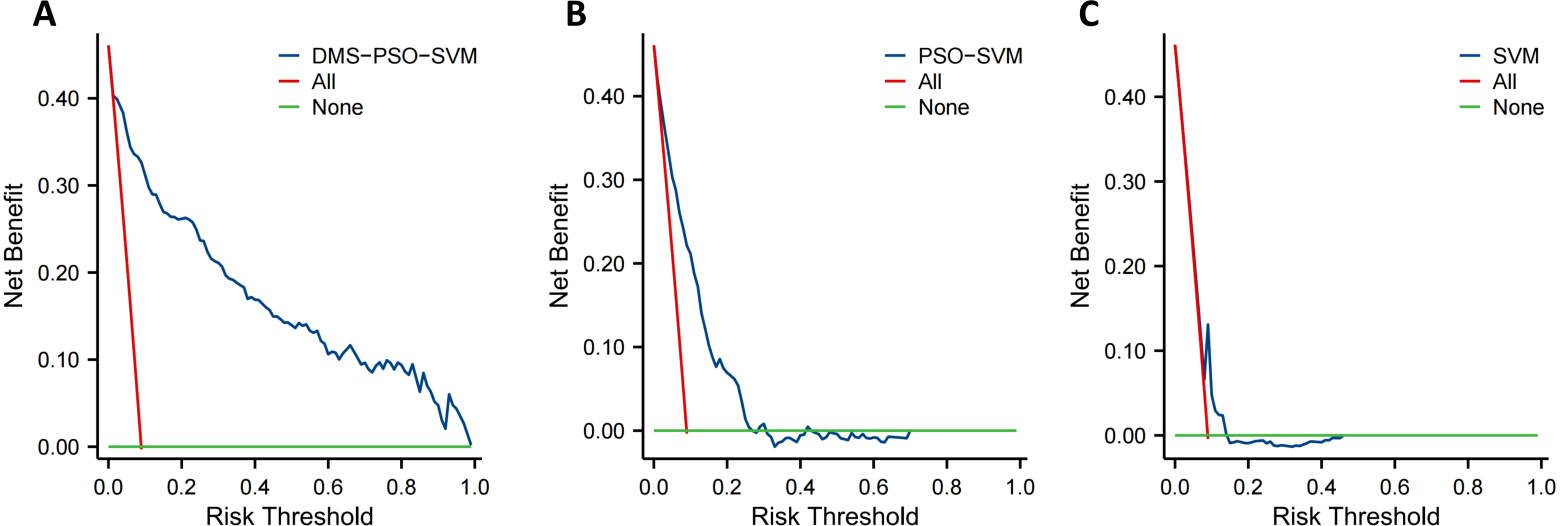
Decision curve analysis of nasopharyngeal carcinoma oligometastatic prediction model. **(A)** Dynamic multi-swarm particle swarm optimization support vector machine. **(B)** Particle swarm optimization support vector machine. **(C)** Support vector machine.

Additionally, to assess clinical applicability under different risk thresholds, decision curve analysis (**Figure 5**) was performed for the three models using the validation set. On the decision curve, the vertical axis denotes net benefit, and the horizontal axis represents the risk threshold at which patients are considered “high-risk,” referencing “treat-all” (assuming all patients develop oligometastasis) and “treat-none” (assuming no patients develop oligometastasis) as comparisons. The decision curve analysis (**Figure 5**) shows that the DMS-PSO-SVM model provides net benefit compared with “treat-all” and “treat-none” strategies across nearly the entire clinically plausible risk threshold range (approximately 0.10–1.00). By contrast, the PSO-SVM model yields positive net benefit only between thresholds of about 0.10–0.30, and the standard SVM model between roughly 0.07–0.14. In **Figure 5A**, when thresholds range from low to moderate, the DMS-PSO-SVM model consistently lies above the “Treat All” and “Treat None” baselines, offering a higher net benefit than other approaches, thereby highlighting its clear clinical decision-making advantage. By comparison, PSO-SVM (**Figure 5B**) remains above “Treat All” and “Treat None” across most threshold ranges but falls slightly below DMS-PSO-SVM in net benefit, while the standard SVM (**Figure 5C**) overlaps or nears the “Treat None” line at certain thresholds, thus conferring only limited net benefit. This result suggests that employing the model’s output probabilities to differentiate high-risk from low-risk patients and make corresponding treatment decisions would offer the greatest net benefit over a broad threshold interval when using DMS-PSO-SVM.

In this study, Based on the principle of maximizing the Youden index in the training set, we determined that the optimal cutoff for the model’s predicted probability is 0.47 (26). Therefore, we adopted a risk-threshold of 0.47 to distinguish high-risk from low-risk patients. Decision curve analysis indicates that at this threshold the DMS-PSO-SVM model maintains a positive net benefit relative to “treat-all” and “treat-none” approaches. Specifically, a net benefit of approximately 0.16 at a 0.47 threshold implies that for every 100 patients evaluated, 16 additional true-positive cases would be identified without increasing false positives. This corresponds to a number needed to treat (NNT) of about 6, meaning that intensified monitoring would correctly capture one extra oligometastasis case for every six patients subjected to enhanced follow-up.

### 3.4 Model interpretation

**Figure 6:**
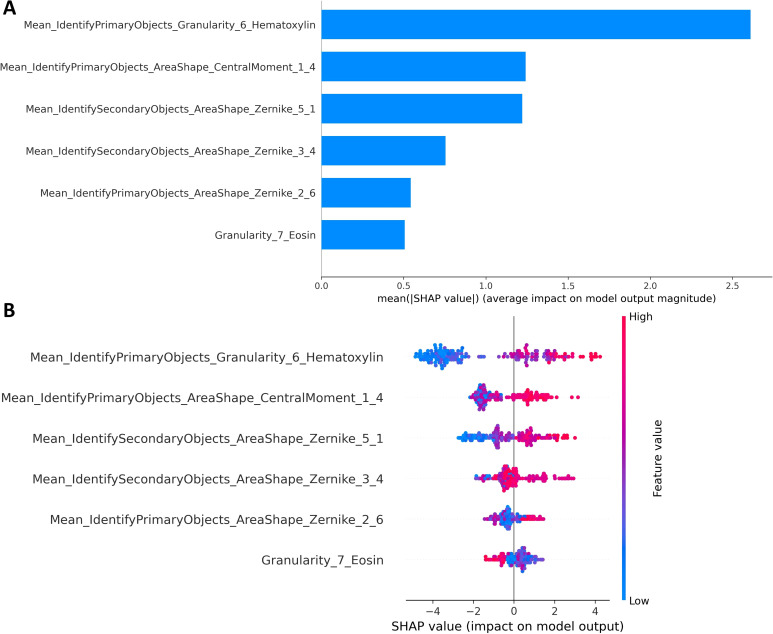
Global and summary SHAP analysis of the DMS-PSO-SVM model. **(A)** Bar plot of the mean absolute SHAP values for each of the six pathomics features, indicating the average magnitude of their contributions to the model output across all validation cases. **(B)** SHAP summary dot plot showing the distribution of individual SHAP values for each feature (horizontal axis) colored by feature value (from low-blue to high-red). Features are ordered by their global importance (mean absolute SHAP value), and the plot illustrates both the direction and strength of each feature’s effect on the predicted risk of oligometastasis.

To understand how individual pathomics measurements drive the DMS-PSO-SVM predictions, we applied the SHAP framework using all 369 training cases as the background distribution. The mean absolute SHAP values across the 93 validation patients (**Figure 6A**) rank Mean_IdentifyPrimaryObjects_Granularity_6_Hematoxylin as the most influential feature, followed by Mean_IdentifyPrimaryObjects_AreaShape_CentralMoment_1_4 and Mean_IdentifySecondaryObjects_AreaShape_Zernike_5_1; least impact is observed for Granularity_7_Eosin. The summary dot plot (**Figure 6B**) further reveals that higher Hematoxylin granularity and larger central moments generally push the predicted risk upward, whereas higher values of Zernike_2_6 and eosin granularity tend to suppress the risk score.

**Figure 7:**
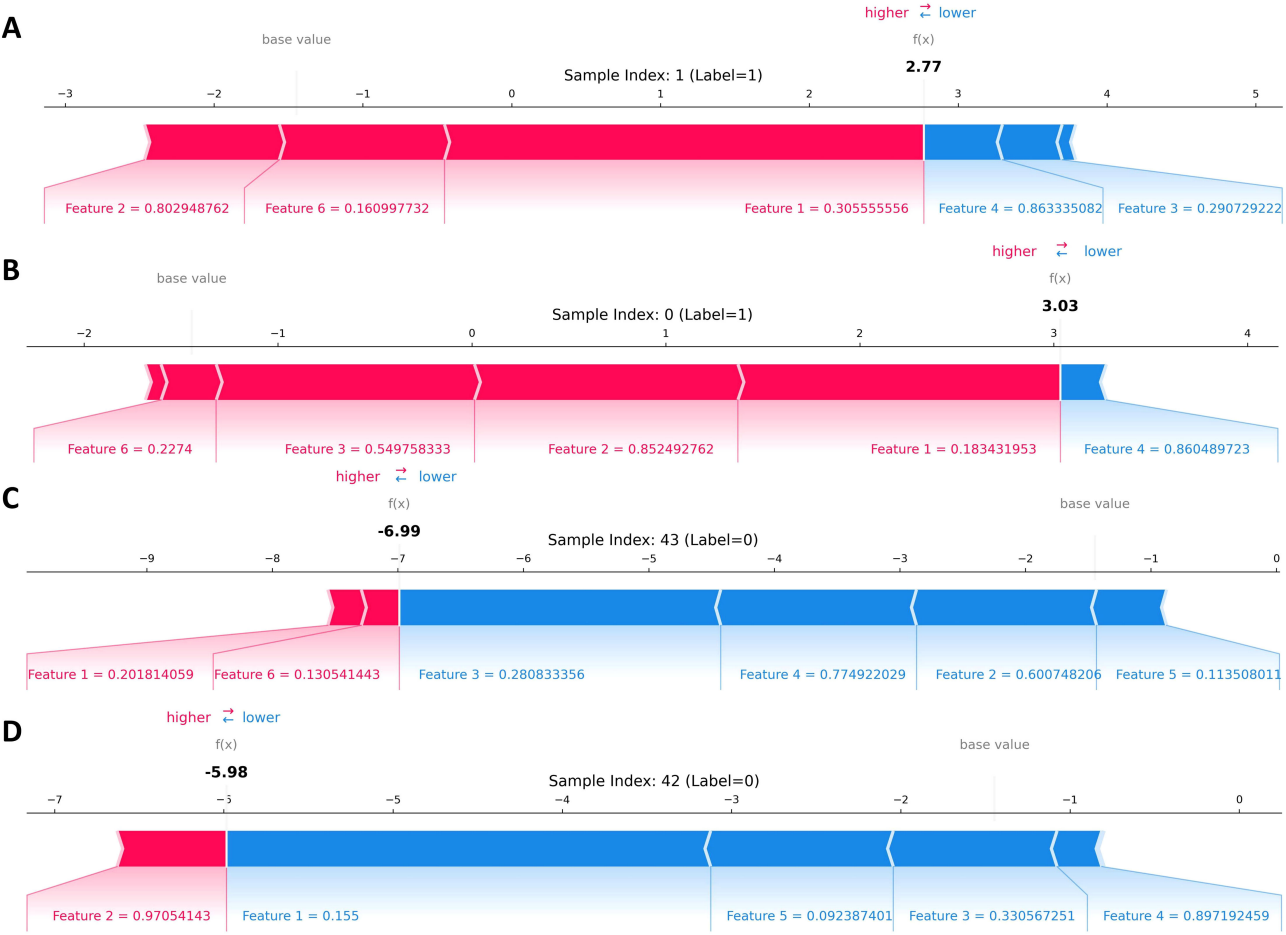
SHAP force plots of four randomly selected patients. **(A, B)** Two patients with the true label of having oligometastases (Label=1) and also classified as having oligometastases in the prediction model. **(C, D)** Two patients with a true label of no oligometastasis (Label=0) and who were classified as having no oligometastasis in the prediction model.

Force plot visualizations for two representative high‐risk patients (both with true labels = 1) show that elevated Granularity_6_Hematoxylin and CentralMoment_1_4 values produce strong positive SHAP contributions that cumulatively raise each individual’s risk prediction well above the base value (**Figures 7A, B**). In contrast, for two low‐risk patients (labels = 0), negative contributions from features such as Zernike_3_4 and Granularity_7_Eosin dominate, driving the model output below the base value and yielding low predicted probabilities (**Figures 7C, D**, bottom panels).

## 4 Discussion

This study primary aim was to evaluate the independent predictive value of quantitative pathomics features. By demonstrating that robust discrimination can be achieved using pathology derived metrics alone, we can more clearly attribute predictive improvements to the pathological biomarkers of oligometastatic potential. Improving an objective method to predict potential oligometastasis before NPC treatment has significant clinical value. This study aimed to enhance the prediction performance for oligometastatic NPC using pathomics and an improved SVM algorithm. By extracting crucial pathomics features and applying dynamic multi-swarm particle swarm optimization to optimize the SVM, the model proposed in this research demonstrated promising performance in the validation set (AUC = 0.866, 95% CI: 0.805–0.928). It outperformed both the SVM optimized by standard particle swarm optimization (PSO-SVM, AUC = 0.721, 95% CI: 0.579–0.862) and the standard SVM (AUC = 0.718, 95% CI: 0.590–0.847). Based on the 47% risk threshold determined in this study, patients with a predicted probability ≥ 0.47 are classified as high-risk by our model. Clinically, these high-risk patients may undergo intensified monitoring and follow-up, such as quarterly MRI or PET/CT examinations during the first two postoperative years to enable early detection of occult metastases. If oligometastasis is identified, corresponding treatment can be administered to delay disease progression and the development of widespread metastases, thereby improving patient survival.

This study revealed a significant difference (P < 0.001) in N Stage prior to treatment between patients who developed oligometastases and those who did not, suggesting that lymph node involvement before treatment may indicate a high-risk profile for oligometastasis and a poorer prognosis. This is consistent with previous reports (27, 28). Earlier studies used radiomic features and clinical variables to construct predictive models for metastasis in NPC. A single-center study by Huang et al. (29), based on multisequence MRI radiomics features, showed promising results in predicting asynchronous single-organ metastasis in NPC (AUC = 0.733). Another study by Peng et al. (30), using PET/CT radiomics features before treatment in 85 NPC patients, employed an SVM to predict distant metastasis, again confirming the capability of image-based features (AUC = 0.829). Compared with prior work, the current study’s model improved AUC by 13% and 4%, respectively, indicating that combining pathomics features with an enhanced predictive algorithm indeed bolsters prediction performance.

In this study, the six pathomics features finally included for model construction are: “Mean_IdentifyPrimaryObjects_AreaShape_CentralMoment_1_4,” “Mean_IdentifySecondaryObjects_AreaShape_Zernike_3_4,” “Mean_IdentifyPrimaryObjects_Granularity_6_Hematoxylin,” “Granularity_7_Eosin,” “Mean_IdentifyPrimaryObjects_AreaShape_Zernike_2_6,” and “Mean_IdentifySecondaryObjects_AreaShape_Zernike_5_1.” Functionally, most of these features involve shape-based quantitative indices (Central Moment, Zernike moments) and texture-based descriptors (Granularity), capturing key information on cellular morphology, structural complexity, and staining properties within the tumor and its microenvironment. Central Moment and Zernike moments, two commonly used shape descriptors, quantify the geometric structure and distribution of cells or tissue regions from different perspectives. Central Moments highlight skewness and dispersion around a central axis, whereas Zernike moments serve as higher-order global shape descriptors relying on orthogonal polynomials to characterize asymmetry, edge complexity, and rotational invariance (31, 32). For tumor sections, such shape metrics help capture nuclear atypia, nuclear-to-cytoplasmic ratio variations, and tissue disarray—all often linked to tumor invasiveness, proliferation, and metastatic propensity. Meanwhile, Granularity features describe the textural grain size and distribution patterns. In H&E-stained sections, the eosin channel in particular correlates with cytoplasmic and stromal staining, indicating factors such as collagen fiber deposition, extracellular matrix alterations, or hyperproliferative tumor cell clusters, which can also affect invasion or metastatic potential (33, 34). The distinction between “IdentifyPrimaryObjects” (e.g., single cells or nuclei) and “IdentifySecondaryObjects” (e.g., cytoplasm, glandular structures, or stromal regions) in CellProfiler reflects different requirements for shape and spatial distribution. Identical shape features like Zernike or Central Moment, if separately calculated for Primary vs. Secondary Objects, might reveal unique changes in each microenvironmental component (35, 36).

At the level of feature screening. To maximize robustness and interpretability while mitigating overfitting in our high-dimensional, moderate-sample-size study, we employed a sequential five-stage feature-selection pipeline. We first retained only those features demonstrating high reproducibility (ICC > 0.75), thereby ensuring consistency across repeated image processing (20). A subsequent Mann-Whitney U test (P < 0.05) excluded features lacking significant association with oligometastatic status (21). To address multicollinearity, we removed one member of any feature pair with Spearman |ρ| > 0.90, preserving original feature identities rather than transforming them into abstract axes (22). The minimum redundancy maximum relevance (mRMR) algorithm then prioritized variables that combined maximal relevance to the outcome with minimal inter-feature redundancy (23). Finally, LASSO regression with an L1 penalty and five-fold cross-validation refined this set further by shrinking weak coefficients to zero, thus yielding a parsimonious, well-generalizing predictive model (24). Unlike principal component analysis, which projects all measurements into orthogonal components (PC1, PC2, etc.) that lack direct histopathological interpretation, our approach maintains a clear link between each selected metric and known tissue phenomena (37). Recursive feature elimination (RFE) was also considered, but its instability in small to moderate cohorts and the absence of intrinsic effect-size estimates limited its appeal. By contrast, the combined mRMR-LASSO strategy offers a transparent, two-tiered filter-wrapper framework: mRMR efficiently narrows the candidate pool to the most informative features, and LASSO assigns each retained feature a non-zero coefficient that directly reflects its predictive contribution. This balance of interpretability and performance makes our pipeline particularly well suited to pathomics-driven risk modeling.

Compared with earlier investigations, this study introduced dynamic multi-swarm particle swarm optimization at the algorithm level, which affords global search capabilities and multi-swarm collaboration to counteract the pitfalls of high-dimensional parameter spaces in pursuit of local optima. This provides a more robust parameter-optimization method for SVM. The multi-swarm parallel search and periodic regrouping mechanism allow the model to maintain high sensitivity while simultaneously preserving specificity, thereby still achieving favorable generalizability in the context of complex pathomics features. Unlike previous work—primarily focused on radiomics features and clinical variables—this study zeroes in on the micro-level heterogeneity in pathology slides, leveraging the high-throughput data on fine-grained textures, cell morphology, and spatial distribution from digital pathology. The results validate pathomics’ potential in prognostic prediction. This research offers clinical value and innovation in several aspects. First, extracting pathomics features from digital pathology slides provides a more direct reflection of tumor microenvironment and cellular changes, circumventing resolution and subjective interpretation issues potentially present in imaging alone (9). Second, for pathomics’ high-dimensionality and possibly multimodal distribution, adopting DMS-PSO for SVM parameter tuning, together with cross-validation to forestall overfitting, ultimately yields a model featuring high prediction accuracy and robustness. Moreover, the model has shown stability across different datasets (training and validation sets), lending more concrete external feasibility for future clinical application.

Deploying a predictive model based on pathomics in everyday clinical settings requires addressing multiple challenges. It is essential to implement a comprehensive digital pathology workflow that includes calibrating whole slide scanners and standardizing staining protocols so that pathomics features can be extracted with consistency. Effective tissue segmentation and quantitative feature computation demand specialized image analysis software, for example CellProfiler or similar platforms (38). To make the model accessible within routine practice, it could be provided as an independent graphical application or incorporated as a plugin into existing digital pathology viewers (39). Interpreting model outputs and applying risk thresholds will rely on close collaboration among clinicians, pathologists and data engineers, supported by targeted training sessions. Seamless integration with clinical information systems depends on robust interfaces between the laboratory information system and the electronic health record, allowing pathology images, feature data and prediction results to populate patient records automatically for multidisciplinary review. Once deployed, a quality assurance and performance monitoring regime must be established. This would involve regular re-validation using contemporary local specimens, tracking any drift in discrimination and calibration metrics, and updating model parameters or decision cutoffs as needed to maintain optimal performance (40).

Although this study achieved promising efficacy in the validation set with the current sample size. However, this study has certain limitations. First, no additional variables from laboratory analyses (e.g., EBV DNA levels, immunological indicators) or multi-omics data (genomics, proteomics, radiomics) were incorporated into the analysis. Future work could explore the integration of these modalities with pathomics to further improve predictive accuracy. Second, although this single-center retrospective study collected a substantial sample size at one center, its data collection and patient characteristics remain relatively constrained. Given that different institutions exhibit significant variability in tissue staining protocols (e.g., H&E reagent manufacturers, staining durations), digital slide scanning hardware, and image-preprocessing workflows (such as color deconvolution and image enhancement), these factors can affect the stability of pathomics feature extraction and the predictive performance of the model. Moreover, baseline pathological characteristics and the tumor microenvironment differ across regions and patient populations, which may further impact the model’s generalizability. To address these limitations, future work will involve collaboration with multiple independent medical centers to collect WSI data generated using diverse staining and scanning platforms and to perform rigorous multicenter external validation.

## 5 Conclusion

Using pathomics features extracted from digital pathology images, coupled with an improved SVM algorithm, this study constructed a predictive model for post-treatment oligometastatic risk in nasopharyngeal carcinoma. The model demonstrated notable advantages in discrimination, calibration, and clinical utility. With continuous advances in pathological data acquisition and algorithmic refinement, pathomics-based predictive models are expected to play an increasingly important role in precision medicine for NPC, providing valuable assistance in early intervention and personalized therapy for high-risk oligometastatic patients.

## Data Availability

The raw data supporting the conclusions of this article will be made available by the authors, without undue reservation.
