# Supplementary material for: Construction of an oligometastatic prediction model for nasopharyngeal carcinoma patients based on pathomics features and dynamic multi-swarm particle swarm optimization support vector machine
Source: Front Oncol. 2025 Jun 19;15:1589919. doi: 10.3389/fonc.2025.1589919 (PMC12222258; doi:10.3389/fonc.2025.1589919)
Supplement: Supplementary file 1 [file Supplementaryfile1.pdf]

Supplementary File 1:

Table S1: Baseline information of Patients

Characteristics	Oligometastatic Patients (n=95)	Patients Without oligometastatic (n=367)	P value
Gender, n (%)			0.157
Male	61 (13.2%)	263 (56.9%)	
Female	34 (7.4%)	104 (22.5%)	
Age, median (IQR)	49 (45, 56)	51 (46, 57)	0.161
T Stage, n (%)			0.316
T3	36 (7.8%)	167 (36.1%)	
T1	14 (3%)	33 (7.1%)	
T2	29 (6.3%)	105 (22.7%)	
T4	16 (3.5%)	62 (13.4%)	
N Stage, n (%)			< 0.001
N2	45 (9.7%)	210 (45.5%)	
N1	10 (2.2%)	48 (10.4%)	
N3	36 (7.8%)	67 (14.5%)	
N0	4 (0.9%)	42 (9.1%)	
AJCC, n (%)			< 0.001
IV	65 (14.1%)	125 (27.1%)	
III	30 (6.5%)	208 (45%)	
II	0 (0%)	23 (5%)	

Characteristics	Oligometastatic Patients (n=95)	Patients Without oligometastatic (n=367)	P value
I	0 (0%)	11 (2.4%)	
Drinking, n (%)			0.034
Yes	46 (10%)	134 (29%)	
No	49 (10.6%)	233 (50.4%)	
Hypertension, n (%)			0.799
No	82 (17.7%)	313 (67.7%)	
Yes	13 (2.8%)	54 (11.7%)	

Supplementary File 2:

Table S2: Important pathomics features:

Feature Name
Mean_IdentifyPrimaryObjects_AreaShape_CentralMoment_1_4
Mean_IdentifySecondaryObjects_AreaShape_Zernike_3_4
Mean_IdentifyPrimaryObjects_Granularity_6_Hematoxylin
Granularity_7_Eosin
Mean_IdentifyPrimaryObjects_AreaShape_Zernike_2_6
Mean_IdentifySecondaryObjects_AreaShape_Zernike_5_1

Supplementary File 3:

DMS-PSO-SVM

Search Space:

$$C \in [10^{-3},\ 10^{3}]$$

$$\gamma \in [10^{-4}, 1]$$

Optimal Hyperparameters:

$$C = 15.81$$

$$\gamma = 0.0024$$

Supplementary File 4:

SVM (Grid-Search Strategy)

Search Space:

$$C \in [0.1,1,10,50,100,200,500,1000]$$

$$\gamma \in [10^{-3}, 10^{-2}, 10^{-1}, 10^{-0.5}, 10^{0}, 10^{0.5}]$$

Optimal Hyperparameters:

$$C = 79.26$$

$$\gamma = 0.08$$

Supplementary File 5:

Performance Comparison of DMS-PSO-SVM and mainstream CNN Models:

All experiments in this comparison used the same set of 512 × 512-pixel RGB patches extracted from diagnostic H&E whole-slide images. Prior to training, each input image was normalized by subtracting the ImageNet channel means and dividing by the ImageNet standard deviations. To enhance robustness against staining and scanning variability, we applied on-the-fly data augmentation including random horizontal flips, rotations of up to ±15°, color jitter (brightness, contrast, saturation), and random crops preserving at least 90% of the original area.

This study adapted four main stream standard CNN architectures: ResNet-50, VGG16, InceptionV3, and DenseNet-121were initialized with ImageNet pretrained weights. During fine-tuning, the first 80% of each network's layers were frozen, while the remaining

convolutional blocks and a newly appended classification head (a 512-unit fully connected layer with dropout p=0.5, followed by a softmax output) were trained. The classification head parameters were randomly initialized using Xavier uniform initialization. All models used the Adam optimizer; the learning rate was set to 1×10^{-3} for the classification head and 1×10^{-4} for the unfrozen backbone layers. Each model was trained with a batch size of 16 for up to 100 epochs. Validation AUC was evaluated at the end of each epoch: if it did not improve for 10 consecutive epochs (patience = 10), training was terminated early. Additionally, if the validation loss did not decrease for 5 consecutive epochs, the learning rate was multiplied by 0.5. All CNN training and evaluation were performed on a single NVIDIA RTX 5090 GPU (32 GB).

Table S2: Model performance of different models on the validation set

Model	AUC	Accuracy	Sensitivity	Specificity
ResNet-50	0.610	0.614	0.503	0.720
VGG-16	0.725	0.708	0.619	0.711
Inception-V3	0.743	0.730	0.607	**0.850**
DenseNet-121	0.816	0.791	**0.825**	0.807
SVM	0.718	0.724	0.726	0.693
PSO-SVM	0.721	0.733	0.728	0.769
DMA-PSO-SVM	**0.866**	**0.820**	0.819	0.846

Note: Bolded text indicates the best model for the metric.

In the validation cohort, the DMS-PSO-SVM model demonstrated superior discrimination (AUC = 0.866) and overall accuracy (82.0%). However, its highest sensitivity (true-positive rate) was slightly exceeded by DenseNet-121 (82.5% vs. 81.9%), and its highest specificity (true-negative rate) was marginally outperformed by Inception-V3 (85.0% vs. 84.6%). Traditional SVM and PSO-SVM models exhibited lower AUCs (0.718 and 0.721, respectively), while ResNet-50 and VGG-16 showed comparatively weaker performance across all metrics. Taken together, these findings indicate that although certain CNN architectures may achieve the best results on individual measures, the DMS-PSO-SVM model provides the most balanced performance profile, particularly with respect to discrimination and overall classification accuracy.
